# Artificial intelligence and twice-exceptional (2e) learners in educational settings: a scoping review of an emerging evidence base (2020–2026)

**DOI:** 10.3389/fpsyg.2026.1927003

**Published:** 2026-09-21

**Authors:** Yasir Alsamiri

**Affiliations:** Department of Education, Islamic University of Madinah, Madinah, Saudi Arabia

**Keywords:** adaptive learning, artificial intelligence, giftedness, learning disabilities, scoping review, systematic review, twice-exceptional

## Abstract

Twice-exceptional (2e) students are intellectually or creatively gifted and also have one or more disabilities, and they require provision that develops talent and addresses disability at the same time. Artificial intelligence (AI) is increasingly promoted as a route to personalized instruction, and its adaptive character appears conceptually well suited to the heterogeneous profiles of 2e learners. Whether that promise has been examined empirically with this population has not been established. This scoping review asked whether and how AI technologies have been reported in educational research involving 2e learners between 2020 and the search date in 2026. Following PRISMA-ScR, a population-anchored search was conducted in ERIC in August 2026 and supplemented by hand searching of a themed journal issue and by reference checking, returning 65 records of which five were assessed at full text. Two studies met the eligibility criteria, both published in 2025 and both in the same themed journal issue: the evidence base is emerging rather than absent, but it is very small and has begun through editorial intervention rather than sustained accumulation. A further group of records sat close to the eligibility boundary and is reported in full so that the limits of the evidence are visible. Across the included studies, sample sizes are small, outcome measurement is uneven, and no study examines whether AI systems that adapt to observed performance interact with the masking that defines twice-exceptionality. An affordance–risk framework is proposed, with each proposition explicitly labeled as hypothesized rather than demonstrated, together with a research agenda for a field that is beginning rather than absent.

## 1 Introduction

Twice-exceptional (2e) students are those who are intellectually and/or creatively gifted and who also have a disability such as attention-deficit/hyperactivity disorder (ADHD), autism spectrum disorder (ASD), dyslexia, dysgraphia, dyscalculia, or another learning disability. They are best characterized by the interaction between the two labels rather than by either in isolation: strengths and difficulties coexist, interact, and are frequently mistaken for one another. Provision for these learners therefore has to promote talent and meet disability-related needs simultaneously, not in separate and sequential programes.

One well-documented consequence of that interaction is the masking effect. Giftedness compensates for and obscures a disability, while the disability depresses measured performance and conceals the giftedness. Masking contributes to delayed identification, misdiagnosis and inappropriate placement, and it is particularly difficult to detect through the standardized, single-dimensional assessments that many school systems rely on Kranz et al. (2024), Maddocks (2020), and Yssel et al. (2020). Recent systematic work on identification methods for 2e learners reaches similar conclusions about the limits of conventional psychometric approaches (Maki et al., 2026; Rizzo et al., 2025). In school and out-of-school settings alike, teachers report being underprepared to recognize 2e profiles and lacking institutional support for responding once they do (Alsamani et al., 2023, 2026; Alsamiri and Alsamani, 2026; Gierczyk and Hornby, 2021).

Attention to artificial intelligence in education has grown rapidly over the same period. The term covers a set of technologies that differ materially from one another, and the distinctions matter for this review. Generative AI and conversational agents produce text, images or dialogue in response to prompts. Intelligent tutoring systems model a learner's state and sequence instruction accordingly. Adaptive learning platforms adjust difficulty or pacing along a progression. Learning analytics infer patterns from behavioral and performance data, often for identification or early warning. AI-supported decision systems present those inferences to teachers or administrators as a basis for action. Each of these has a different purpose, uses different evidence, generates different inferences, and produces outputs that are consumed in different ways. Grouping them together as “AI” obscures precisely the differences that determine whether a given system is likely to help or harm a 2e learner.

The appeal for this population is easy to state. Because 2e profiles are heterogeneous and asynchronous, the prospect of challenge in areas of strength alongside scaffolding in areas of difficulty is attractive, and personalization is the capability AI is most often marketed on. The difficulty is that most educational AI infers learner ability from observed performance, and observed performance is exactly what masking distorts. A system that adapts faithfully to what a 2e learner appears able to do may reproduce the masking rather than penetrate it, reading disability-driven errors as evidence of low ability rather than of concealed high ability. Broader work on algorithmic fairness makes the same general point: models trained on typical distributions perform least well on the atypical cases at the distribution's edges, which is where this population sits.

Reviews adjacent to this question have begun to appear. Ronksley-Pavia et al. (2025b) mapped generative AI for neurodivergent school students and identified a body of work in which gifted and twice-exceptional learners feature, but as one group among several. Alsamani and Alsamiri (2026) mapped AI across gifted and talented education more broadly. Neither took the intersection of giftedness and disability as its unit of analysis. What has not been established is whether, and in what form, research reports AI technologies used with explicitly identified 2e samples, what outcomes are measured, and what risks are documented. This review addresses that question directly, and treats the shape of the answer—including its sparseness—as a substantive finding.

### 1.1 Aim

The aim of this review is to map whether and how artificial intelligence has been reported in educational research involving twice-exceptional learners between 2020 and the 2026 search date, to characterize the technologies, populations and outcomes represented, and to identify the gaps that follow.

### 1.2 Research questions

Consistent with a scoping design, the questions ask whether and how a literature exists rather than presupposing that it does:

- RQ1: whether and in what forms have AI technologies been reported in educational research involving twice-exceptional learners?
- RQ2: which categories of AI technology are represented, and in which educational contexts and populations?
- RQ3: what educational, behavioral, cognitive or social outcomes, if any, have been reported, and how were they measured?
- RQ4: what challenges, ethical issues or implementation barriers are reported or anticipated in this literature?
- RQ5: what gaps remain, and what should a future evidence base establish?

### 1.3 Objectives

To identify and characterize reported AI applications involving 2e learners; to chart the technologies, samples, designs and outcomes represented; to distinguish demonstrated findings from conceptual propositions; to document records that approach but do not meet eligibility, so that the boundary of the evidence is visible; and to derive a research agenda.

## 2 Materials and methods

### 2.1 Review design and reporting

This is a scoping review. It is designed and reported in accordance with the PRISMA extension for scoping reviews (PRISMA-ScR; Tricco et al., 2018), and the completed PRISMA-ScR checklist is provided. A scoping design was selected because the objective is to map the extent, range and nature of an emerging literature and to identify gaps, rather than to appraise or pool effects across a defined body of trials. The review is described as a scoping review throughout; the terms “systematic review” and “systematic scoping review” are not used to describe this work, and PRISMA 2020 is cited only where the underlying reporting conventions are shared.

### 2.2 Protocol

The research questions, eligibility criteria, search strategy, and screening procedure were specified in an internal protocol before searching began. The protocol was not registered in a public registry. No deviations from the protocol occurred during the conduct of the review.

### 2.3 Eligibility criteria

Eligibility was defined using the PICOS framework (Table 1). Two levels of evidence were distinguished prospectively. Primary eligible studies are empirical studies reporting a defined AI technology used with an explicitly identified 2e sample and at least one reported educational outcome. Conceptual and design papers at the same intersection—frameworks, design proposals and methodological pieces that address AI and 2e learners without reporting learner-level outcomes—were charted separately, because a scoping review of an emerging field that excluded them would misrepresent the shape of the literature. Contextual literature on twice-exceptionality without an AI element was retained at a third level for interpretation only, and is not counted as included evidence at either of the first two levels.

**Table 1:** Eligibility criteria defined using the PICOS framework.

Component	Inclusion	Exclusion
Population (P)	Twice-exceptional students of any age or educational level: identified or described as gifted or high-ability and also having one or more co-occurring disabilities or neurodevelopmental conditions (e.g. specific learning disabilities, ADHD, ASD).	Gifted-only or disability-only samples; undifferentiated neurodivergent samples in which 2e learners are not separately identified or reported.
Intervention/concept (I)	A defined AI technology: generative AI or conversational agents, intelligent tutoring systems, adaptive learning platforms, learning analytics, machine learning, or AI-supported decision systems.	Digital or assistive technology with no AI component; AI referred to only in passing without specification of the technology.
Comparator (C)	None required; where present, traditional instruction, non-AI technology, or an alternative intervention.	—
Outcomes (O)	Any reported learning-related outcome: academic achievement, engagement, cognitive or behavioral performance, socio-emotional outcomes, accessibility, identification or assessment accuracy, or reported acceptability and experience.	No reported outcome of any kind (for the primary level only; such records are charted at the conceptual level).
Study design (S)	Primary level: quantitative, qualitative, mixed-methods, experimental, quasi-experimental and observational studies. Conceptual level: frameworks, design papers and methodological pieces at the AI × 2e intersection.	Editorials and commentaries without developed argument; existing reviews, which were screened for reference harvesting and discussed as related work rather than charted as studies.

Records had to be published in English between 2020 and the search date. Peer-reviewed journal articles were eligible for the primary and conceptual levels. Conference proceedings, dissertations, reports and other gray literature were not eligible for those levels but were searched separately as a sensitivity check (Section 2.6), because AI applications in education frequently appear in conference and practice literature before reaching journals.

### 2.4 Information sources and search strategy

The database search was conducted in ERIC (eric.ed.gov) on 20 August 2026 using the search term 2e twice-exceptional, with the Peer Reviewed Only limiter applied. No date limiter was applied at the interface; the 2020–2026 eligibility window was applied at the screening stage instead, so that the number of records excluded on publication date is visible in the flow diagram rather than hidden inside the search. The search returned 62 records.

The search term was deliberately anchored on the population rather than on the intersection. Twice-exceptionality is indexed inconsistently and the literature is small, so a population-first search followed by screening for an AI component retrieves more of the relevant material than a narrower intersectional string, at the cost of a lower precision rate. The consequence is visible in the flow: most excluded records are studies of twice-exceptional learners in which no AI technology is present.

One included record was published in a themed issue of the Journal of Advanced Academics devoted to artificial intelligence in gifted education. That issue was therefore hand-searched article by article, which identified three further records. Reference lists of eligible records and of adjacent reviews were also checked. Because 2026 was an incomplete publication year at the time of searching, coverage of that year is partial and extends only to the search date.

The abbreviation 2e is searched only as a bound term within the phrase above. Entered as a standalone token it is tokenized by several platforms and retrieves large volumes of irrelevant material containing e-prefixed words such as e-learning and e-portfolio; this was verified during strategy development and the term was constrained accordingly. The study identification and selection process is summarized in Figure 1.

**Figure 1:**
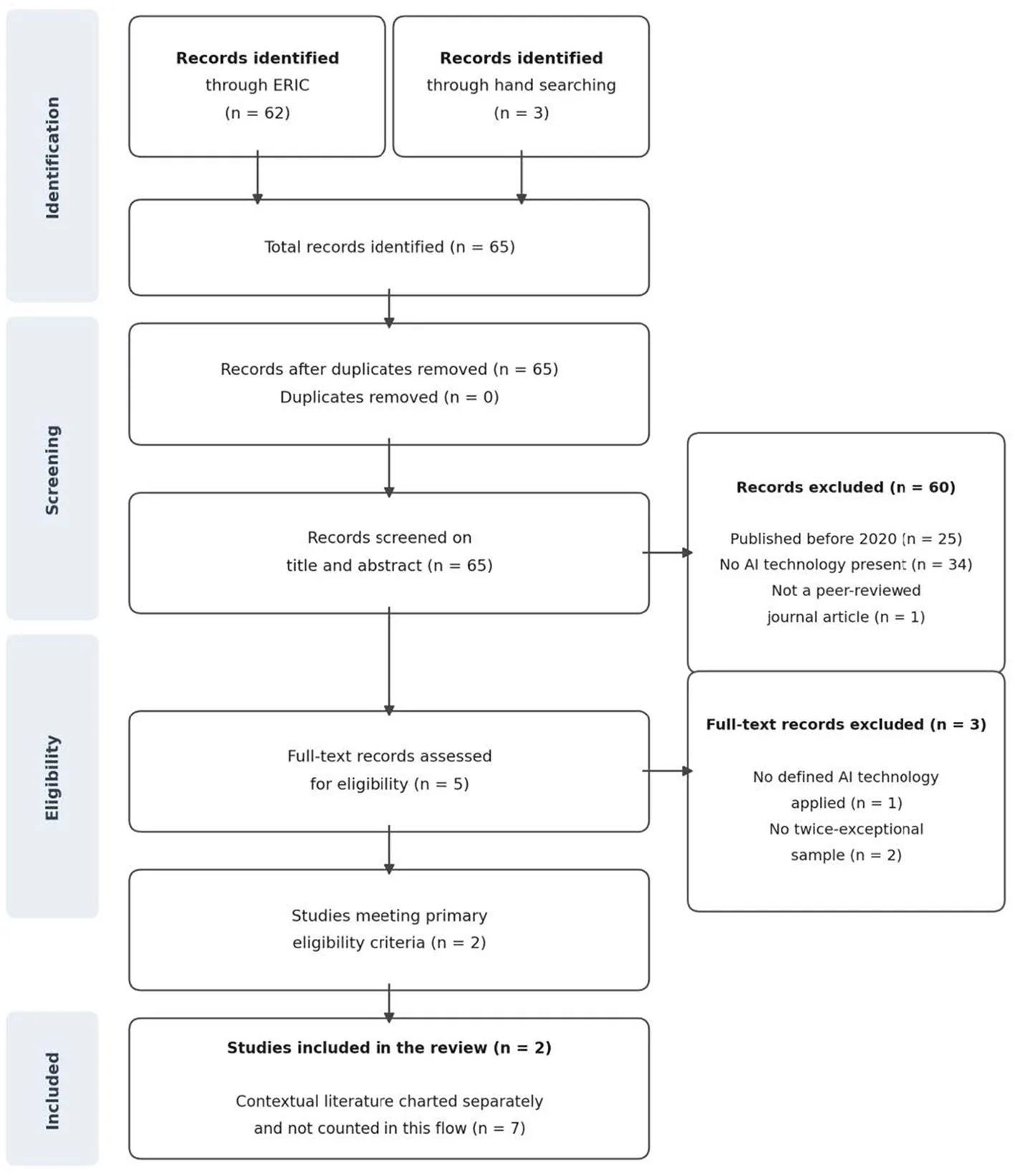
PRISMA-ScR flow diagram of identification, screening, eligibility and inclusion.

### 2.5 Selection process

Title and abstract screening and full-text eligibility assessment were conducted by a single reviewer (YA). Records were exported directly from ERIC and screening decisions were recorded in a spreadsheet. Single-reviewer screening is acknowledged as a limitation in Section 4.4.

Every record assessed at full text was assigned one exclusion reason, and each record appears exactly once in the flow diagram. Records that met all criteria except one are reported individually in Table 2 rather than absorbed into a summary count, so that readers can see how close the boundary cases came and judge the eligibility rule for themselves.

**Table 2:** Records assessed at full text that approached but did not meet eligibility.

Record	Relevance	Reason not eligible
Akdeniz et al. (2025)	Compares AI-generated and human-generated questions aligned to Bloom's revised taxonomy, evaluated by gifted-education experts.	Gifted education generally; no twice-exceptional sample.
Ecker et al. (2025)	Proposes a four-dimensional framework for integrating AI tutoring into gifted programming across affect, content, process and product.	Gifted education generally; no twice-exceptional sample and no reported outcomes.
Lewis and Milam (2026)	Praxis paper on dual differentiation for 2e learners in teacher preparation, engaging AI literature.	Twice-exceptional focus but no defined AI technology applied and no learner outcomes.
(Ronksley-Pavia et al. 2025b)	Scoping review of generative AI for neurodivergent school students, 2020–2024, in which gifted and twice-exceptional learners appear among the populations discussed.	A review rather than a primary study; screened for reference harvesting and discussed as related work.
Alsamani and Alsamiri (2026)	Scoping review mapping AI across gifted and talented education, identifying 26 eligible studies.	A review rather than a primary study; twice-exceptionality is not the unit of analysis. Discussed as related work.
Hulsey et al. (2023)	Compares behavioral engagement across gifted, disability, 2e and general-education profiles using a national dataset, framed around masking and identification accuracy.	Twice-exceptional sample but secondary analysis of survey data with no AI technology applied.

### 2.6 Sensitivity search of conference and gray literature

Because educational AI develops faster than journal publication cycles, the restriction to peer-reviewed journal articles carries a risk of under-representing the field. The search was run with the Peer Reviewed Only limiter applied, and one conference proceedings volume returned by the search was excluded on that basis. A dedicated sensitivity search of conference proceedings and gray literature was not conducted, and this is stated as a limitation in Section 4.4 rather than presented as a completed check.

### 2.7 Data charting and synthesis

A charting form was developed and piloted before use, capturing bibliographic details, country, sample description and 2e identification method, AI technology and category, educational purpose, study design, outcomes and measures, and reported limitations. Charted data are summarized descriptively.

The affordance–risk framework in Section 3.6 was constructed through a structured mapping procedure rather than informal reasoning. Documented characteristics of 2e learners were extracted from the contextual literature and listed. Each characteristic was then paired systematically with each of the five AI categories defined in Section 1, and for every pair two questions were asked: what mechanism in that technology could plausibly benefit a learner with this characteristic, and what mechanism in the same technology could plausibly disadvantage them. Propositions were retained where a mechanism could be named and traced to a documented characteristic; they were discarded where the link rested only on general enthusiasm about personalization. Because the included studies do not test these propositions, every entry is labeled as hypothesized, and that labeling is maintained in the Discussion.

## 3 Results

### 3.1 Selection of sources of evidence

The ERIC search returned 62 records and hand-searching added a further three, giving 65 records identified. No duplicates were present. All 65 were screened at title and abstract, and 60 were excluded: 25 published before 2020, 34 containing no AI technology, and one that was not a peer-reviewed journal article. Five full texts were assessed. Two met the primary eligibility criteria and three were excluded, each with a single stated reason; all three are reported individually in Table 2. The complete screening log, giving a decision and where applicable a reason for every one of the 65 records.

The contextual literature summarized in Table 3 was identified from reference lists rather than from this search. It is not counted in the flow diagram and does not appear in the screening log, consistent with its status as interpretive background rather than included evidence.

**Table 3:** Contextual literature used for interpretation (not included evidence).

Source	Type	Characteristic documented
Kranz et al. (2024)	Systematic narrative review	Masking of dyslexia by giftedness and the resulting failure of single-dimension identification.
Maki et al. (2026)	Systematic review	Identification methods for 2e learners and their limitations.
Rizzo et al. (2025)	Systematic review	Distinctive characteristics of 2e learners across six domains of functioning.
Alsamani et al. (2023)	Qualitative	Teacher interpretation of 2e strengths and difficulties.
Alsamani et al. (2026)	Qualitative	Perceived barriers to identifying and supporting 2e students, including training and resourcing.
Alsamiri and Alsamani (2026)	Qualitative	Professional development needs in inclusive settings.
Karakaş Misir and Thomas (2026)	Interpretive systematic review	Policy, practice and socio-emotional context.

### 3.2 Characteristics of included studies

The eligible literature is small, very recent, and concentrated in a single outlet (Table 4). Both included studies were published in 2025 in the Journal of Advanced Academics, and both appear in the same themed issue on artificial intelligence in gifted education. That concentration is itself informative: it indicates a field in which activity has begun through a deliberate editorial intervention rather than through sustained independent accumulation.

**Table 4:** Characteristics of included studies.

Study	Level/design	Sample	AI technology	Purpose and reported outcomes
Mahmoudi-Dehaki and Nasr-Esfahani (2025)	Primary; explanatory sequential mixed methods with a control group	12 twice-exceptional bilingual adolescents with motor dysgraphia and ADHD, with above-average verbal memory relative to normative data	Human–AI hybrid tutoring (intelligent tutoring system with human instructor)	Writing instruction over 32 sessions. Reported gains in handwriting fluency (speed and legibility), orthographic coding, fine motor control and composition. Focus groups reported high acceptability, attributed to adaptive delivery, immediate feedback and socio-emotional support.
Ronksley-Pavia et al. (2025a)	Primary; small-scale experimental study without human learner participants	Evidence-informed synthetic learner profiles constructed to represent twice-exceptional and multi-exceptional neurodivergent students	Generative AI (large language models)	Development of GenAI-informed approaches for teachers to rehearse pedagogy. Reported promise in generating adaptive, personalized approaches, alongside difficulty in securing consistent, context-appropriate and unbiased outputs; the authors underline the necessity of human oversight.

Two features of this body of work are immediately apparent. First, it is almost entirely a product of the last 2 publication years, which is consistent with the wider growth of generative AI in education rather than with sustained attention to twice-exceptionality. Second, the technologies represented are unevenly distributed: generative AI and tutoring systems dominate, while learning analytics and AI-supported identification—the applications most directly relevant to the masking problem—are essentially unrepresented in studies with 2e samples.

### 3.3 Records at the eligibility boundary

Several records were relevant to the review question but fell outside eligibility for a specific and stateable reason. Reporting them individually serves two purposes: it makes the eligibility rule auditable, and it shows readers where the field is active even where the evidence does not yet meet the standard this review set.

### 3.4 Answers to the research questions

#### 3.4.1 RQ1 and RQ2 (whether, and in what forms)

AI has been reported in educational research involving 2e learners, but only recently and in a small number of studies. Generative AI predominates, followed by tutoring systems used in combination with human instruction. Adaptive learning platforms, learning analytics and AI-supported identification systems are effectively absent from studies with explicitly identified 2e samples, despite being the categories most often invoked when the potential of AI for this population is discussed. Contexts are narrow: individual or small-group instruction rather than classroom-level deployment, and no system-level or policy-level implementation was identified.

#### 3.4.2 RQ3 (outcomes)

Where outcomes are reported, they concentrate on writing and related skills, together with acceptability and learner experience. Measurement is uneven. Sample sizes are very small, and one of the eligible studies works with synthetic rather than human learner profiles, which limits what can be inferred about effects on learners. No study reports identification or assessment accuracy outcomes for 2e learners, and none reports socio-emotional outcomes as primary endpoints. No replication was identified.

#### 3.4.3 RQ4 (challenges and ethics)

The included studies raise the consistency and appropriateness of AI outputs, the risk of bias in generated material, and the necessity of human oversight. What they do not examine is the interaction between AI systems and masking, which the contextual literature identifies as the defining feature of this population. The teacher-preparedness gap documented across the 2e literature (Alsamani et al., 2026; Alsamiri and Alsamani, 2026) is directly relevant here, since AI outputs about a 2e learner have to be interpreted by educators who report being underprepared to recognize these profiles in the first place.

#### 3.4.4 RQ5 (gaps)

The principal gap is not the absence of a literature but its shape. The applications most likely to affect 2e learners at scale—analytics-driven identification and adaptive systems keyed to observed performance—are the least studied with this population. Section 4.5 sets out the resulting agenda.

### 3.5 Contextual literature and sensitivity check

Contextual sources on twice-exceptionality were retained to support interpretation. They are not included evidence and were not used to answer RQ1 to RQ3; they inform only the characteristics side of the affordance–risk mapping described in Section 2.7. Their concentration is stated openly: several are authored or co-authored by the present author, and the framework in Section 3.6 draws additionally on the wider 2e literature cited throughout this review in order not to rest on a single research group.

No dedicated sensitivity search of conference proceedings or gray literature was conducted. One conference proceedings volume was returned by the database search and excluded under the peer-review criterion. The possibility that relevant activity exists outside the peer-reviewed journal literature therefore remains open, and is recorded as a limitation rather than as a tested question.

### 3.6 Hypothesized affordance–risk framework

The framework below is derived by the procedure set out in Section 2.7. Every proposition in it is hypothesized. None is demonstrated by the included studies, and none should be cited as an established effect for this population. The framework's function is to specify what a future evidence base would need to test, and to make each proposition falsifiable enough to be worth testing.

## 4 Discussion

### 4.1 An emerging rather than absent evidence base

This review set out to establish whether and how AI has been reported in educational research involving twice-exceptional learners. The answer is that a literature exists, that it is very recent, and that it is thin in a specific and consequential way. Characterizing it as an absence would misstate the position; characterizing it as an evidence base would overstate it. What can be said within the boundaries of this review is that a small number of eligible studies published between 2020 and the 2026 search date report a defined AI technology used in connection with explicitly identified 2e learners, that their samples are small, and that the categories of AI most likely to shape these learners' educational trajectories at scale are the least represented among them.

That is a claim bounded by the review's parameters, and it is stated as such. It does not establish that no other work exists, and in particular it does not extend to non-English publications, to work published after the search date, or to research in which 2e learners are present within undifferentiated neurodivergent samples but not separately identified or reported. The last of these is likely to be the largest reservoir of relevant activity, since neurodiversity-focused AI research is expanding quickly and rarely disaggregates by exceptionality profile.

### 4.2 Masking and performance-keyed personalization

The most consequential observation from this mapping is a mismatch that no included study examines. Twice-exceptionality is defined by discrepant performance and by the mutual concealment of ability and disability. Contemporary educational AI, across nearly all its categories, infers learner state from observed performance. The more faithfully a system adapts to what a 2e learner appears able to do, the more precisely it may encode the masking, treating disability-driven errors as evidence of limited ability rather than of ability that is being obscured.

If that is right, the intuition that adaptivity is a natural fit for 2e heterogeneity may be inverted, and the population most frequently named as a beneficiary of personalized AI may be among the most exposed to its failure modes. This is a hypothesis, not a finding, and this review provides no evidence either way. It is stated here because it is testable, because the test is straightforward to design, and because the studies that would settle it have not been done.

### 4.3 Why technology category matters

Treating AI as a single object obscures the point above. A generative system asked to produce differentiated material and a learning-analytics model asked to flag students for assessment carry entirely different risks for a masked profile. The generative system's failure mode is content: material that is inappropriate, inconsistent or biased, and which a teacher can in principle inspect. The analytics model's failure mode is inference: a judgement about a learner that is difficult to inspect, is produced at scale, and may enter an identification pathway with institutional weight behind it. The second is the more serious risk for this population, and it is the one about which the least is known, because the eligible studies cluster in the first category. Implications should therefore be drawn per technology category rather than for AI in general, and the framework in Table 5 is organized accordingly.

**Table 5:** Hypothesized affordances and hypothesized risks, by AI category and documented 2e characteristic.

Documented 2e characteristic	AI category	Hypothesized affordance	Hypothesized risk
Masking of disability by giftedness and vice versa	Learning analytics; AI-supported identification	Fine-grained behavioral and performance data may expose discrepant patterns that aggregate scores conceal, prompting earlier referral.	Models fitted to typical performance distributions may treat a masked profile as unremarkable, reproducing under-identification with the added authority of an algorithmic output.
Asynchronous development	Adaptive learning platforms	Difficulty may be adjusted independently by domain, allowing enrichment and scaffolding to run concurrently.	Systems built on a single difficulty progression may average across a discrepant profile, delivering material that is simultaneously too easy and too hard.
Need for simultaneous enrichment and accommodation	Generative AI; multimodal tools	Advanced content may be delivered together with accommodation within one activity rather than through separate provision.	Few systems are designed for this dual objective, and outputs optimized for accessibility may reduce cognitive demand at the same time.
Socio-emotional vulnerability	Conversational agents; intelligent tutoring systems	Low-stakes, patient interaction may reduce the exposure associated with visible difficulty in front of peers.	Affective inference is unreliable; systems may misread frustration in a learner whose expressive profile is atypical, and may displace human contact for learners who already report isolation.
Highly individualized profiles	All categories	Personalisation may accommodate strengths and difficulties that group-level provision cannot.	Training data drawn from general populations under-represents this group, so personalisation is least reliable precisely where it is most needed.
Limited teacher expertise in recognizing 2e profiles	AI-supported decision systems	Decision support may surface learners warranting further assessment who would otherwise be overlooked.	Outputs interpreted by underprepared educators may be over-trusted, and an algorithmic recommendation may harden an existing judgement rather than challenge it.

### 4.4 Strengths and limitations

The review's strengths are a narrow and clearly specified eligibility rule, a boundary-case table that makes that rule auditable, and full reporting of search strategies for reproduction. Several limitations qualify the findings.

- The review was restricted to English-language publications, which excludes relevant work in other languages.
- Coverage of 2026 extends only to the search date and is therefore partial for that year.
- Journal-only eligibility at the primary and conceptual levels may under-represent a fast-moving field; the sensitivity search in Section 3.5 was conducted to test this.
- The review searched a single database, ERIC, supplemented by hand searching and reference checking. Subscription databases including Scopus, Web of Science and PsycINFO were not accessible. This is the review's most significant limitation: a single-database search cannot support a claim of comprehensive coverage, and records indexed elsewhere but not in ERIC will have been missed.
- No dedicated sensitivity search of conference proceedings or gray literature was conducted, so the effect of the peer-review restriction on the results is untested.
- Screening was conducted by a single reviewer, which introduces a risk of missed records that a dual-reviewer process would reduce.
- Contextual sources are concentrated in a small number of research groups including the present author's, which is stated openly and mitigated by drawing the framework on the wider literature as well.

### 4.5 Implications and research agenda

The immediate implication for practice is that claims about what AI can do for twice-exceptional learners currently rest on a very small number of small studies, and that the confident language used in product and policy discussion is not matched by the evidence. The agenda that follows is specific:

- Intervention studies with explicitly identified 2e samples, adequately powered, reporting educational, cognitive and socio-emotional outcomes, and stating the identification method used to establish twice-exceptionality.
- Direct tests of whether learning analytics detect masked profiles that standardized assessment misses, and of whether adaptive systems attenuate or amplify masking over time. This is the central empirical question and it is currently unaddressed.
- Design and evaluation frameworks in which enrichment and accommodation are treated as simultaneous objectives rather than as opposite ends of a single difficulty axis.
- Evaluation of algorithmic fairness for this population specifically, including the representativeness of training data and error rates for discrepant profiles.
- Studies of teacher interpretation: how educators read, weigh and act on AI-generated outputs about 2e learners, given the documented preparedness gap.
- Reporting practice in neurodiversity-focused AI research that disaggregates 2e learners rather than absorbing them into undifferentiated samples, without which this intersection will remain difficult to map.

## 5 Conclusion

This scoping review mapped whether and how artificial intelligence has been reported in educational research involving twice-exceptional learners between 2020 and the 2026 search date. A small, recent and unevenly distributed literature was identified: generative AI and tutoring applications are represented, while the analytics and identification systems most likely to affect these learners at scale are not. Because twice-exceptionality is defined by discrepant and masked performance, and because educational AI infers learner state from observed performance, the possibility that personalization reproduces masking rather than penetrating it is the question the field most needs to answer, and it is the one on which no evidence yet exists. The affordance–risk framework offered here is hypothesized throughout and is intended as a specification for that work rather than as a summary of what is known. The task is not to synthesize an evidence base that already exists, but to build one deliberately, with these learners' distinctive profiles in view.

## Data Availability

The raw data supporting the conclusions of this article will be made available by the authors, without undue reservation.

## References

[B1] Akdeniz H. Clark T. Roberts J. L. (2025). Can AI generate questions aligned with Bloom's taxonomy? a framework for gifted education to support teachers. J. Adv. Acad. 36, 671–694. doi: 10.1177/1932202X251349917

[B2] Alsamani O. A. Alsamiri Y. A. (2026). Artificial intelligence in gifted and talented education: a scoping review. Front. Psychol. 17:1898467. doi: 10.3389/fpsyg.2026.1898467 42591964 PMC13463290

[B3] Alsamani O. A. Alsamiri Y. A. Alabdulwahab R. A. (2026). Twice-exceptionality in primary schools: teachers' perceived barriers to identifying and supporting 2e students. Acta Psychol. 262:106031. doi: 10.1016/j.actpsy.2025.106031 41319421

[B4] Alsamani O. A. Alsamiri Y. A. Alfaidi S. D. (2023). Elementary school teachers' perceptions of the characteristics of twice-exceptional students. Front. Educ. 8:1150274. doi: 10.3389/feduc.2023.1150274

[B5] Alsamiri Y. A. Alsamani O. A. (2026). Teachers' perceptions of professional development needs for identifying and supporting twice-exceptional students in inclusive schools in Saudi Arabia. Educ. Sci. 16:366. doi: 10.3390/educsci16030366

[B6] Ecker J. Eckert G. Cummings E. (2025). Augmenting gifted education through artificial intelligence: a four-dimensional framework for AI tutoring integration. J. Adv. Acad. 36, 879–896. doi: 10.1177/1932202X251356326

[B7] Gierczyk M. Hornby G. (2021). Twice-exceptional students: review of implications for special and inclusive education. Educ. Sci. 11, 85. doi: 10.3390/educsci11020085

[B8] Hulsey D. B. Moten T. R. Hebda M. R. Sulak T. N. Bagby J. H. (2023). Using behavioral engagement measures of multiple learning profiles to recognize twice-exceptional students. Gift. Child Today 46, 13–24. doi: 10.1177/10762175221131055

[B9] Karakaş Misir S. Thomas M. (2026). Gifted but misunderstood? an interpretive systematic review of gifted education policy, practice, and socio-emotional experience in England. J. Intell. 14:34. doi: 10.3390/jintelligence14030034 41892876 PMC13028215

[B10] Kranz A. E. Serry T. A. Snow P. C. (2024). Twice-exceptionality unmasked: a systematic narrative review of the literature on identifying dyslexia in the gifted child. Dyslexia 30:e1763. doi: 10.1002/dys.1763 38232949

[B11] Lewis K. D. Milam M. E. (2026). Powerful learning: dual differentiation for twice-exceptional learners. J. Adv. Acad. 37, 147–175. doi: 10.1177/1932202X251396237

[B12] Maddocks D. L. S. (2020). Cognitive and achievement characteristics of students from a national sample identified as potentially twice exceptional. Gift. Child Quart. 64, 3–18. doi: 10.1177/0016986219886668

[B13] Mahmoudi-Dehaki M. Nasr-Esfahani N. (2025). Supporting 2e bilingual students with motor dysgraphia and ADHD in writing: efficacy and acceptability of human–AI hybrid tutoring. J. Adv. Acad. 36, 640–670. doi: 10.1177/1932202X251348549

[B14] Maki K. E. Dougé M. Rubenstein L. D. Ridgley L. M. DeShon L. (2026). Seeing both sides: a systematic review of identification methods for twice-exceptional learners. J. Educ. Gift. 49, 246–273. doi: 10.1177/01623532261459140

[B15] Rizzo L. Pinnelli S. Minnaert A. (2025). Twice-exceptional students: a systematic review to outline the distinctive characteristics through a multidimensional lens. Front. Educ. 10:1696805. doi: 10.3389/feduc.2025.1696805

[B16] Ronksley-Pavia M. Nguyen L. Wheeley E. Rose J. Neumann M. M. Bigum C. . (2025b). A scoping literature review of generative artificial intelligence for supporting neurodivergent school students. Comput. Educ. Artif. Intell. 9:100437. doi: 10.1016/j.caeai.2025.100437

[B17] Ronksley-Pavia M. Ronksley-Pavia S. Bigum C. (2025a). Experimenting with generative AI to create personalized learning experiences for twice-exceptional and multi-exceptional neurodivergent students. J. Adv. Acad. 36, 601–639. doi: 10.1177/1932202X251346349

[B18] Tricco A. C. Lillie E. Zarin W. O'Brien K. K. Colquhoun H. Levac D. . (2018). PRISMA extension for scoping reviews (PRISMA-ScR): checklist and explanation. Ann. Intern. Med. 169, 467–473. doi: 10.7326/M18-0850 30178033

[B19] Yssel N. Neumeister K. Burney V. H. (2020). “Students with special needs who are gifted and talented (twice exceptional),” in Oxford Research Encyclopedia of Education (Oxford: Oxford University Press). doi: 10.1093/acrefore/9780190264093.013.1218

